# Effectiveness of communications in enhancing adherence to public health behavioural interventions: a COVID-19 evidence review

**DOI:** 10.1098/rsta.2023.0129

**Published:** 2023-10-09

**Authors:** Simon N. Williams, Kimberly Dienes, Jemma Jaheed, Jamie K. Wardman, Judith Petts

**Affiliations:** ^1^ School of Psychology, Swansea University, Vivien Tower, Singleton Park Campus, Swansea, Wales SA2 8PP, UK; ^2^ Department of Medical Social Sciences, Feinberg School of Medicine, Northwestern University, Chicago, IL 60208, USA; ^3^ Manchester Centre for Health Psychology, University of Manchester, Manchester M13 9PL, UK; ^4^ School of Business, Leicester University, Leicester LE2 1RQ, UK; ^5^ University of Plymouth, Plymouth PL4 8AA, UK

**Keywords:** COVID-19, health communication, non-pharmaceutical interventions, behavioural interventions, adherence

## Abstract

Health communication has relevance for virtually every aspect of health and well-being, including disease prevention. This review explored the effectiveness of communications in enhancing the adoption of or adherence to behavioural interventions (non-pharmaceutical interventions (NPIs)) related to COVID-19. The review takes the UK as a case study and focuses on self-reported behaviours (e.g. social distancing). It also reviews the psychosocial determinants of adherence. Searches were conducted using PubMed, Scopus, CINAL, ASSIA and iCite databases. Eleven thousand five hundred records were identified and 13 were included in the final sample. Included studies suggest that NPI adoption or adherence was generally high, and communication had significant impacts, with key themes including clarity and consistency, trust and control. Based on the evidence in this review, features of effective communication in the context of NPI adoption or adherence are (i) information should be conveyed clearly and conflicting (mixed) messages should be avoided; (ii) information should be conveyed by trusted sources (e.g. health authorities) and (iii) communication should strike a balance between being authoritative but avoiding language seen as controlling (e.g. ‘you must’). Future research should prioritize quantitative, experimental and longitudinal study designs, that focus specifically on communication as an intervention, and which measure behaviour.

This article is part of the theme issue 'The effectiveness of non-pharmaceutical interventions on the COVID-19 pandemic: the evidence'.

## 1. Introduction

Health communication has relevance for virtually every aspect of health and well-being, including disease prevention [1]. In the context of public health emergencies like the COVID-19 pandemic, effective risk communication can enable people to take informed decisions to protect themselves and others, reduce illness and save lives [2]. During the COVID-19 pandemic, rapid, effective communication was needed to convey accurate information around non-pharmaceutical interventions (NPIs), like facemask wearing, self-isolation and physical distancing from governments, health authorities, scientists and other key health actors to the public. There is a growing body of evidence looking at adherence to COVID-19 public health behavioural interventions (hereafter NPIs). This evidence from the UK suggests that overall adherence to NPIs was generally high, particularly in the early stages of the pandemic, even for the more challenging, ‘higher cost’ NPIs, like lockdowns [3,4] There are a number of factors that predict adherence [3,5], but communication is a critical one [6]. It has been suggested that all intervention efforts to change behaviours are communicative acts [1,7]. In a sense, as far as NPIs are concerned, communication can be seen as a ‘meta-intervention’. Meta-interventions are procedures that can be designed to change an audience's behaviour with respect to the preventive interventions themselves, including their participation in them [8]. Even if evidence demonstrates that a given NPI, like facemasks, are efficacious in reducing virus transmission in clinical or experimental settings, they are unlikely to be effective in real-world settings if they are not communicated well. Put simply, NPIs are unlikely to be effective, if people are not aware of, or do not understand, whether, when, where or how to do them [7].

Effective communication is therefore cited as a key component of NPIs for COVID-19 [9,10]. ‘Health communication’ is however difficult to define, precisely because it entails such a broad and inclusive set of constructs or principles [11]. Health communication, as the *exchange of information*, can be seen to entail a number of interrelated components, including (i) a *message* (what information is being communicated and how is it being framed), (ii) a communication channel (which media is the message being sent across), (iii) a communication source (who is sending the message) and (iv) an audience (who is receiving or interpreting the message) [12]. One useful definition is ‘the use of communication strategies to inform and influence individual and community decisions that enhance health’ [13]. This definition identifies two of the key purposes of health communication: informing (or educating) and influencing (or persuading)—two key functions of behaviour change interventions [14,15].

Communication interventions also do not fall into a ‘social vacuum’, with a range of social and cultural factors and channels heavily influencing how communicated information is received and processed [1,11,16]. Indeed, disentangling communication from the changing context and nature of the policies and laws being communicated (especially in a rapidly changing public health emergency) is challenging. Furthermore, one increasingly important role of communication has been to combat the COVID-19 ‘infodemic’, characterized as an overabundance of information or misinformation (inaccurate or misleading information, including but not limited to COVID-19 conspiracy theories) shared online via social and other media [17]. This underscores the need for public health communication to strive to be accessible, actionable by decision-makers, credible and trusted, relevant, timely and understandable [2]. In the context of public health emergencies like COVID-19, it has been suggested that an effective communication strategy is ‘a two-way process that involves clear messages, delivered via appropriate platforms, tailored for diverse audiences, and shared by trusted people’ [7,16]. Research has further identified that the implementation of interventions operates within a system of key intersecting behavioural components, including capability, opportunity and motivation, which shape their effectiveness [14]. These behavioural conditions have been highlighted as especially important for COVID-19 interventions when considered in the context of implementing change during pandemic restrictions [18].

Some general criticisms of research on behaviour change interventions have been identified. First, although media health campaigns have been seen to have small measurable short-term effects on behaviour change [19], evidence on sustaining behaviour change (maintenance) is relatively limited [20]. Second, the intention–behaviour gap, where people do not necessarily ‘follow through’ on their intentions by acting, is a well-established known challenge for public health communication [21]. For instance, a recent review on health authorities’ health risk communication to the public during pandemics found that evidence on protective behaviour was lacking (with most studies focused on content or engagement with communication media) [22]. This reflects a wider historic concern by critics highlighting the difficulties often found in pointing to health risk communication ‘success stories’, or interventions that have effectively translated evidence-based principles into action [23].

Building on these broader understandings, for the purposes of this review, we define communication as the conveyance of information around NPIs employed by the UK during the COVID-19 pandemic, including but not limited to social media, print media and television. Also, to maintain high policy relevance and for pragmatic reasons it was decided that the review should be tightly focused on the United Kingdom rather than a broader study of the international evidence from different, confounding social and cultural settings. This evidence review therefore screened and synthesized literature looking at the impact of communication specifically on *behaviours and actions* related to NPIs in the United Kingdom.

### (a) Research question

What is the best-available evidence about the effectiveness of communication in improving adoption of, or adherence to, NPIs in relation to COVID-19 in non-healthcare, community-based settings?

The sub-questions of this review are as follows:

1. What is the best-available evidence about the types of communication strategies being used to encourage adoption of, or adherence to, NPIs in relation to COVID-19 in non-healthcare, community-based settings?
2. What is the best-available evidence about which types of communication strategies are the most effective at encouraging adoption of, or adherence to NPIs in relation to COVID-19 in non-healthcare, community-based settings?
3. In the context of communication, what is the best-available evidence about the psychosocial determinants of the adoption of, or adherence to NPIs in relation to COVID-19 in non-healthcare, community-based settings?

## 2. Methods

Candidate studies were retrieved from the following databases, with search strings developed by the research team in consultation with a Medical Librarian (Swansea University) and following review by substantive experts in the field of COVID-19 and public health communication: (i) PubMed, (ii) Scopus, (iii) CINAL, (iv) ASSIA and (v) iCite's COVID database for preprints.

The search was limited to studies focused on, or including the UK, and was not restricted according to certain study types or designs. The date range for candidate studies was set at 1 January 2020, to coincide with the emergence of COVID-19 as a global pandemic, to 31 December 2022. We used database-specific adaptations of the following generic search string (see electronic supplementary material for details on database-specific search strings) box 1:

Box 1.
**Search strategy.**

(‘COVID 19’ OR ‘COVID’ OR ‘sars cov 2’ OR ‘Sars’ OR ‘severe acute respiratory syndrome coronavirus 2’ OR ‘Coronavirus*’ OR ‘Corona Virus*)

AND

(‘communicat*’ OR ‘health knowledge’ OR ‘health education’ OR ‘campaign*’ OR ‘access to information’ OR ‘mass communication’ OR ‘mass medi*’ OR ‘messag*’ OR ‘information shar*’ OR ‘information trans*’ OR ‘guidance’ OR ‘public engag*’ OR ‘public understand*’ OR ‘misinform*’ OR ‘disinform*’ OR ‘infodemic’ OR ‘conspirac*’ OR ‘alert’ OR ‘press conferenc*’ OR ‘social media’ OR ‘broadcast*’ OR ‘health literacy’ OR ‘nudg*’ OR ‘prompt*’ OR ‘persua*’ OR ‘attitud*’ OR ‘intention*’)

AND

(United Kingdom’ OR ‘UK’ OR ‘England’ OR ‘English’ OR ‘Wales’ OR ‘Welsh’ OR ‘Scotland’ OR ‘Scottish’ OR ‘Northern Ireland’ OR ‘Northern Irish’ OR ‘British’ OR ‘Britain)

**Figure 1.**
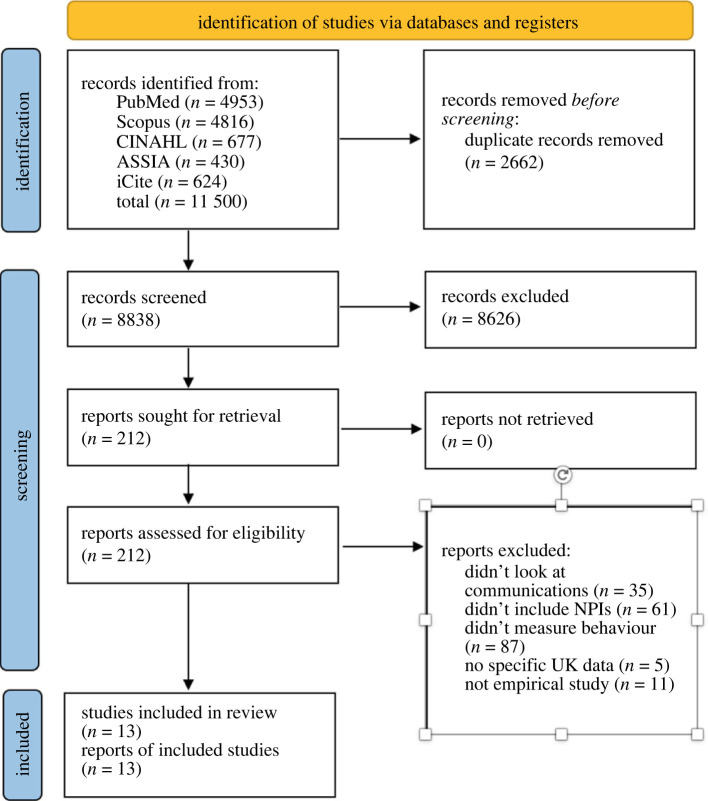
PRISMA 2020 Flow Diagram.

Following the removal of duplicates, returned citations were screened independently by two reviewers (S.N.W. and K.D.) at the title/abstract levels. Full papers were screened by four reviewers (S.N.W., J.J., J.K.W. and K.D.) with two reviewers independently reviewing each paper. Disagreements were first additionally reviewed by a third reviewer, before being discussed by the research team and resolved by consensus. Separate inclusion/exclusion criteria were applied at each stage of screening—first, the title/abstract screen then the full paper review (see box 2 and figure 1 (PRISMA flow diagram)).

Box 2.
**Inclusion and Exclusion criteria were used during the review.**

Inclusion criteria, title/abstract screen; all criteria must be met for inclusion:

— English language studies
— reporting on COVID-19 alone, or COVID-19 and other respiratory infectious diseases
— reporting on communication as a method of increasing the uptake of, or adherence to, non-pharmaceutical interventions (NPIs) for COVID-19
— community-based setting (e.g. schools, daycares, residential settings, retail, restaurants, gyms and other athletic facilities, bars, workplaces, public parks, etc.)

Exclusion criteria, title/abstract screen; any one criterion is grounds for exclusion:

— non-English language studies
— does not report on COVID-19
— does not report on any NPI related to COVID-19
— any type of healthcare setting (e.g. long-term care, acute care, inpatient clinics, emergency department) are to be excluded; if relevant to our topic, they will be tagged according to setting for potential future use
— systematic reviews, narrative reviews and other evidence syntheses are to be excluded

Inclusion criteria, full-text screen:

— studies must report empirical data
— study designs: trials and experimental studies, including randomized and non-randomized designs; observational studies (cohort, case–control and cross-sectional studies); qualitative studies
— studies must include a focus on communication strategies or interventions to encourage adoption of or adherence to NPIs; i.e. studies must include focus on behaviour or action in relation to NPIs (not intention or attitudes to NPIs)
— studies must include data related to the United Kingdom (either as a case study or as a multi-country comparative study including the United Kingdom)

Exclusion criteria, full-text screen:

— studies focused on e-learning in general, not learning about COVID NPIs
— studies not related to uptake of/adherence to COVID behaviours/NPIs—including studies focused only on intentions, willingness or attitudes towards NPIs uptake/adherence
— studies not focused on communication (as an intervention or strategy—see definition above)
— studies focused on vaccination uptake
— studies not including the UK as an empirical case
— studies that discuss impact of NPIs on communication and interaction (e.g. facemasks, distancing on remote communication) (rather than vice-versa)
— studies focused on the psychosocial impact of NPIs and COVID communications (including media coverage)
— studies on telehealth or health-related communication between healthcare professionals and patients (not specifically related to COVID)
— studies that used communication media (e.g. Twitter analysis) to investigate something related to COVID-19 other than NPIs

Data extraction was performed by four members of the research team (S.N.W., J.J., J.K.W. and K.D.) and summarized in a data extraction table, with each summary being checked for accuracy by one other team member. Disagreements or inconsistencies in extracted data were discussed as a research team and resolved by consensus. Studies were assessed for Risk of Bias (observational) or study quality (qualitative). For observational studies, we used ROBINS-I (Intervention) (a tool to evaluate the tendency for non-randomized studies to differ systematically from the results expected from a randomized trial, according to a number of domains, including participant selection, confounding, measurement etc.) [24]) (It should be noted that communication as an intervention does not lend itself as well to assessment using ROBINS-I as other NPIs, and so RoB categorizations should be treated with caution). For qualitative studies, we used the CASP (Critical Appraisal Skills Programme) checklist for qualitative studies (which includes 10 questions for assessing the validity of the method and results) [25]. Observational studies were initially graded for risk of bias, and qualitative studies for study quality, by one reviewer, with one other researcher checking for accuracy; issues or inconsistencies were discussed and resolved by consensus by the research team. Extracted data were reviewed and a narrative synthesis was performed, following the approach set out by Popay *et al*. [26]. Guided by the review objectives (research questions) extracted data were analysed to draw out a number of key themes across studies.

## 3. Results

A total of 11 500 records were identified through the search strategy. Following the removal of duplicates, 8838 records were screened for eligibility using study titles and abstracts. The full texts of 212 studies were then assessed for eligibility. Thirteen papers were included in the final sample (figure 1, PRISMA flow diagram).

Overall, included studies were very heterogeneous in terms of their methods, objectives, content and focus (e.g. the types of communications explored and the types of NPIs included—see below). The 13 studies included zero randomized controlled trials (RCTs) or experimental studies; five observational studies (four cross-sectional survey; one longitudinal survey); seven qualitative studies and one mixed-method study (using cross-sectional survey and qualitative interviews). Table 1 summarizes the methods and findings of the studies included in the review.

**Table 1.** Table summarizing studies selected to report on the effectiveness of communication on COVID-19 NPIs uptake or adherence.

reference	location	type of study	study scope	key findings related to communications and NPIs
Allington *et al*. [27]. Erratum in: *Psychol Med*. 2021; **51**:1770	UK	observational (cross-sectional)	summary: assessing relationships between Covid-19 conspiracy beliefs, social media and Covid-19 health-protective behaviours	information source: there was a small positive relationship between following health-protective behaviours and information sources, specifically: traditional media U(N1 = 1610, N2 = 563) = 478 174.0, *p* < 0.046, 95% CI (0.50–0.56) but a larger effect specifically for TV and radio, U(N1 = 1601, N2 = 561) = 481 068.5, *p* = 0.006, 95% CI (0.51–0.56)
			study period: study 1: 3–7 April 2020	there was a large negative relationship between use of social media as a source of knowledge about COVID-19 and engagement in health-protective behaviours, U(N1 = 1603, N2 = 563) = 342 191.5, *p* < 0.001, 95% CI (0.35–0.41) and a weaker negative relationship between use of friends and family as a source of knowledge about COVID-19 and engagement in all health-protective behaviours, U(N1 = 1601, N2 = 560) = 240 145.0, *p* < 0.001, 95% CI (0.40–0.47)
			Study 2: 1–3 April 2020	conspiracy theories: there was a strong negative relationship between holding one or more conspiracy beliefs and engagement in all health-protective behaviours, *p* < 0.001, 95% CI (0.29–0.47)
			Study 3: 20–22 May 2020	
			sample: study 1. self-selected sample (*n* = 949) (age <18 years)	
			study 2 (*n* = 2250) & study 3 (*n* = 2254) stratified random (quota) samples, (age 16–75 years)	
			NPIs studied: several health-protective behaviours; handwashing (less than 20s); social distancing (2 m rule); social gatherings (meeting up with friends or family outside of home); having friends or family visit you at home; going to work or outside despite having symptoms that could be coronavirus	
			communications types/sources: COVID-19 conspiracy theories on social media as a source of information also: broadcast media (major newspapers and/or TV channels including online)	
			psychosocial constructs//frameworks: study assessed relationships between different Covid-19 conspiracy beliefs and Covid-health-protective behaviours	
Juanchich *et al*. [28]	UK	observational (cross-sectional surveys)	summary: three studies testing for correlations between COVID-19 conspiracy theories and health-protective behaviours	correlation analysis showed that conspiracy beliefs were not statistically significantly associated with basic health-protective behaviours
			study period: 20 March 2020–30 April 2020	trust: trust in government was statistically significantly correlated with belief in COVID-19 conspiracy theories (*r* = −0.18, *p* < 0.01)
			sample: study 1: *n* = 302 (female = 68%; White = 86%; modal age, 26–40 = 47%); study 2: *n* = 404 (female = 56%; White = 78%; modal age, 26–40 = 34%); study 3: *n* = 399 (female = 62%; White = 87%; modal age, 26–40 = 44%)	analytical thinking: analytical thinking (cognitive reflection was statistically significantly correlated with belief in COVID-19 conspiracy theories (*r* = −0.31, *p* < 0.01)
			NPIs studied: basic health-protective behaviours (handwashing; hand sanitizing; covering mouth and nose when coughing or sneezing; avoiding touching face; physical distancing; avoiding public gatherings; avoiding public transport)	
			communication types/sources: news consumption habits (public broadcast news)	control: perception of control was mostly not related to conspiracy beliefs; except small, positive correlations between conspiracy beliefs and perception of control for wearing facemasks (*r* = 0.14, *p* < 0.05) and gloves (*r* = 0.11, *p* < 0.05)
			psychosocial constructs//frameworks: trust (in government); cognitive reflection (analytical thinking versus intuition); control (over health-protective behaviours)	
Mahdavian *et al*. [29]	UK	observational (cross-sectional surveys)	summary: explored the impact of factors including personal health risk perceptions, official message quality and source of news, on COVID-19 protective behaviours on COVID-19 protective behaviours	clarity and consistency: a higher level of perceived official message quality (i.e. the clearer the coronavirus-related instructions, recommendations and guidance) was positively associated with enacting more protective behaviour (*t* = 5862, *p* < 0.01)
			study period: 1 April–4 May 2021	information source: results show a significant positive effect of source of news on protective behaviour (*t* = 2355, *p* < 0.05). More frequent use of official news sources (defined as national TV, radio and newspaper as information source for COVID-19 was significantly associated with more protective behaviours
			sample: UK *n* = 830 (female = 51%; modal age group, 18–29 = 20% (ethnicity not stated)	
			NPIs studied: protective behaviours include: face masks; quarantine (lockdown); social distancing, physical distancing; abiding public space; self-isolation	
			communications types/sources: national newspapers, TV and radio	
			psychosocial constructs//frameworks: risk perception; trust	
Nurgalieva *et al*. [30]	includes UK	observational (cross-sectional survey)	summary: survey exploring public attitudes and factors affecting user acceptability and use of contact-tracing apps	news sources (including: online; print; radio and TV) did not have a significant effect on the odds of installing a contact-tracing app
			study period: 12 January and 26 February 2021	trust: higher trust in: politicians (OR = 1.70, CI = 1.28–2.27, *p* = < 0.001), medical doctors and nurses (OR = 1.50, CI = 1.09–2.07, *p* = 0.01), and in the accuracy of the COVID-related news from political party and leaders (OR = 1.39,CI = 1.13–1.73, *p* = < 0.001) all having a positive effect on the odds of installing the contact-tracing app
			sample: total *n* = 871 (female = 66.7%; White = 78.2%; modal age 18–30 = 52.9%; UK = 39.3%)	
			NPIs studied: contact-tracing apps	
			communication types/sources: news sources (including: online; print; radio and TV); and information sources (including: political party and leaders; friends and family; social network influencers; mainstream media; partisan sites; religious organizations; public service or government departments)	
			psychosocial constructs//frameworks: trust (in sources of news/information)	
Legate & Weinstein [31]	includes UK	observational (longitudinal surveys)	summary: study explored whether changes in motivation (autonomous and controlled) predicted time spent at home, and whether motivating aspects of messages related to time spent at home	motivation: increases in autonomous motivation, but not controlled motivation, predicted more time spent at home
			study period: wave 1, mid-March 2020; wave 2, mid-May 2020	perceiving messages as autonomy supportive related to more time spent at home (*B* = 4.72 (s.e. = 1.22), *p* < 0.001, Δ*R*^2^ = 0.02.)
			sample: living alone older adults, *n* = 683 (female = 56%; mean age = 53; UK = 56%)	control: perceiving messages as controlling predicted spending less time at home (*B* = −3.08 (s.e. = 1.15), *p* = 0.008, Δ*R*^2^ = 0.01.)
			NPIs studied: quarantine (stay-at-home)	a mandated message also predicted more time spent at home (*B* = 2.79 (s.e. = 1.00), *p* = 0.005, Δ*R*^2^ = 0.01)
			communications types/sources: public health or governmental agency; personal doctor/physician recommendation News outlet; request by family members and friends	model explains 7% of the variance in staying at home *F*_6, 647_ = 7.93, *R*^2^ = 0.07, adjusted *R*^2^ = 0.06
			psychosocial constructs//frameworks: motivation (autonomous motivation); control; self-determination theory	
Jayes *et al*. [32]	UK	qualitative	summary: a qualitative evaluation of a local COVID-19 walk-in testing site in England, including both attenders and non-attenders	awareness of community testing was found to be generally low
			study period: February–May 2021	information source: around half of those interviewed were initially made aware of Covid testing by communication via their employer. Others heard about Covid testing through word-of-mouth (family/friends or social media) or traditional media. Few people recollected seeing any subsequent promotion or advertising
			sample: *n* = 33 (female = 70%; White = 91%; age, *m* = 49)	clarity and consistency: lack of attendance was found to be due in part to lack of information and some misunderstanding or confusion on who was eligible to attend and why
			NPIs studied: COVID-19 testing	
			communications types/sources: multiple sources (social media, family/friends, employers, advertising, communication via programme stakeholders, e.g. universities and local government)	
			psychosocial constructs//frameworks: none focused on in study	
Leather *et al*. [33]	UK	qualitative	summary: study captured spontaneous reflections, via open-ended survey questions, on adherence to UK government guidance to identify key determinants of COVID-related behaviours	six theoretical domains framework (TDF) was identified; two domains directly related to communications: memory, attention and decision processes; and knowledge. Relevant themes (enablers and barriers to NPI adherence) were
			study period: April 2020	control: government prompts (enabler to NPI adherence) were identified as an enabler and perceptions that autonomy is under threat were identified as a barrier
			sample: representative sample of UK adults, *N* = 2252. (age, *M* = 50.34(s.d. = 17.02); female = 54.8%; White = 93.0%)	trust: enablers were: government sources considered reliable and other information sources, e.g. the World Health Organization or trusted medical professionals were seen as more reliable. A barrier was scepticism about government statistics
			NPIs studied: multiple (UK government's coronavirus guidance, including: staying at home (70% of statements), handwashing (44%), physical distancing (40%))	clarity and consistency: barriers were: interpretation of terms (e.g. ‘essential’); perceived ambiguities in the instructions; and following instructions from other information sources;
			communications types/sources: multiple (inductive study of barriers and enablers to NPI adherence);	
			psychosocial constructs//frameworks: theoretical domains framework (TDF)	
McNulty *et al*. [34]	UK	qualitative	summary: explored using interviews public reactions to the COVID-19 pandemic across diverse ethnic groups	clarity and consistency: participants perceived poor communication around COVID-19 and found government communications to be confusing, also
			study period: June–October 2020	trust: some participants mistrusted news reports and felt that the media had exaggerated or misrepresented COVID-19 statistics
			sample: UK adults from diverse ethnic and cultural backgrounds, *n* = 100 (female = 49%; Asian = 58%, Black = 15%, Mixed = 2%, White = 20%; other = 5%, modal age group, 30–39 = 30%)	control/authority relations: findings also discuss the balance between authority (enforcement of rules) and ‘control-aversion’ (e.g. where a clear rationale for a rule is not communicated, or the public perceive the government as not trusting them)
			NPIs studied: multiple	findings also highlight the need to better communicate scientific uncertainty surrounding COVID-19, use more local, ethnic community-oriented media (e.g. local ethnic minority radio stations) and communicate more widely in multiple languages
			communications types/sources: multiple	
			psychosocial constructs//frameworks: trust (in Government); behaviour change wheel	
Watson *et al*. [35]	UK	qualitative	summary: this was a rapid qualitative evaluation conducted to assess the feasibility of the Southampton COVID-19 Saliva Testing Programme in a community	results from this review found that high levels of communication (open and transparent communication from programme implementers) were important in ensuring community engagement and adherence to PCR Testing programmes
			study period: June–October 2020	clarity and consistency: participants described finding open communication with the programme team reassuring and motivating to engage in testing
			sample: interviews *n* = 77, online focus groups *n* = 20 with *n* = 223 staff, students, pupils and household members from four schools, one university and one community healthcare NHS trust (specific numbers by gender and ethnicity not given, although notes ‘pupils were from diverse ethnic backgrounds’	trust: participants felt that trust was necessary to improve engagement in testing and that trust could be helped by receiving more directed information from credible sources (e.g. university, local government) about the program, including rationale, data protection and test accuracy. The study also discussed how the fact program communications into multiple language was seen as a facilitator to engagement in testing
			NPIs studied: COVID-19 testing (adherence to a weekly community COVID-19 rapid testing programme in schools, GP surgeries and a university)	
			communications types/sources: communication from programme implementers (partnership between local NHS foundation Trust, city council and the university)	
			psychosocial constructs//frameworks: trust	
Williams *et al*. [36]	UK	qualitative	summary: this study assessed public perceptions of non-adherence to Covid-19 protection measures (by self and others)	qualitative data analysis revealed six key themes related to non-adherence to COVID-19 NPIs
			study period: 25 September–13 November 2020	two themes were deemed related to communication
			sample: UK adults, *n* = 51 (female = 49%; White = 63%; modal age group 40–49 = 35%)	cognitive overload: ‘Alert fatigue’ (i.e. receiving too many alerts/messages leading to confusion or ‘overload’ whereby important information can be overlooked, forgotten, or mis-understood)
			NPIs studied: multiple (pandemic protection measures)	trust: trust in government communication (e.g. communication of rules subject to interpretation)
			communications types/sources: multiple/unspecified (communication/information was raised in relation to non-adherence)	clarity and consistency: participants expressed feeling as though measures were often confusing because of the ‘mixed messages' over NPI guidance or rules
			psychosocial constructs//frameworks: alert fatigue (information/cognitive overload); trust (in government communication)	
Woodland *et al*. [37]	UK	qualitative	summary: study explored families' adherence to multiple NPIs during school closures;	adherence was enhanced by
			study period: 16–21 April 2020	clarity and consistency: delivering clear guidance increased (a family's capability to understand what they needed to do to change behaviour);
			sample: parents of children (age < 18), *n* = 30 (female = 67%; White = 67%; age, *m* = 39)	trust: delivering the guidance by a source the parent and child trusts
			NPIs studied: quarantine (stay-at-home); social distancing in public (avoiding others not from own household); physical distancing (2 m rule); hygiene (cleaning home); hygiene (covering coughs/sneezes); hygiene (avoiding touching face)	control/authority relations: families often stated they adhered because they ‘were told to’, especially when communication was by high authority figures (e.g. government or Prime Minister)
			communications types/sources: multiple	
			psychosocial constructs//frameworks: capabilities, opportunities, motivations model of behaviour (COM-B); Theoretical Domains Framework (TDF)	
Wright *et al*. [38]. Erratum in: BMC Public Health. 2022 Mar 24;22(1):581	UK	qualitative	summary: study explored facilitators and barriers to NPI using structural topic modelling (text mining) to extract themes from over 26 000 free-text survey responses from 17 500 UK adults	trust: a lack of trust in government messaging was voiced by 6.5% of responses
			study period: 17–23 December 2020	clarity and consistency: instructions and about what the rules were found to be confusing in 3.45% of responses
			sample: UK adults (age > 18), *n* = 17 500 (female = 74%; White = 97%; modal age group 60 + = 45%)	
			NPIs studied: working from home; social isolation; social distancing (2 m rule) in public (due to the actions of others or environmental constraints); facemasks; sanitizer	
			communications types/sources: multiple (inductive study of barriers and facilitators to NPI adherence to government communications); used structural topic modelling and text-mining techniques to extract themes from free-text survey responses	
			psychosocial constructs//frameworks: trust (in government); COM-B Framework	
Eraso & Hills [39]	UK	mixed-methods (qualitative and observational (cross-sectional)	summary: study used online survey and interviews and regression analysis (quantitative) and framework analysis (qualitative) to explore factors associated with social distancing rule infringements	trust: an additional level of agreement on a 7-point Likert indicating trust in the Government decreases intentional s.d. infringements by 7.4%
			study period: survey: 1–31 May 2020	clarity and consistency: interview participants found government messaging to be confusing with some messages (e.g. ‘stay alert) unclear or subject to interpretation. Interview participants also found messaging to be ‘inconsistent’ or ‘contradictory’
			interviews: 5 August–21 September 2020	
			sample: survey, *n* = 681 (female = 83%; White = 86%; mean age = 43); interviews, *n* = 30 (female = 47%; White = 63%;	
			NPIs studied: social distancing (stay-at-home)	
			communications types/sources: multiple	
			psychosocial constructs//frameworks: trust (in government); perceived behavioural control (self-efficacy and controllability); theory of planned behaviour; the social ecological model	

Among the five observational (survey) studies, using ROBINS-I criteria, three studies were found to have serious risk of bias [28–30] (i.e. the study has important problems, relative to an RCT [24]) and two were found to have moderate risk of bias [27,31] (i.e. sound evidence for a non-randomized study but not comparable to an RCT [24]). Among the qualitative studies, all seven studies [32–38] were deemed high-quality qualitative studies according to CASP criteria.

Overall adherence was generally high. Eight studies [27–29,33,36–39] reported high adherence among participants, with a further two studies [30,31] reporting moderately high adherence. Three studies didn't provide data on levels of adherence per se, only factors related to adherence. For example, one study [27] found that over three-quarters (77.03–79.66%) engaged in one of the three health behaviours studied (hand washing, physical distancing and staying at home) and six in 10 (61.85%) engaged in all three. Studies found average self-rated adherence to be high across multiple protective behaviours, for example 4.36 on a 5-point scale (with 5 being ‘several times per day’ across all studied NPIs [29] or 6.29 on a 7-point scale, where 7 is ‘very much so’ following recommended behaviours). Qualitative studies that explored levels of adherence found perceived self-adherence to be high (if not necessarily perceived adherence by others) [36–39].

### (a) What types of communication and communication strategies have been usedto encourage non-pharmaceutical intervention adoption or adherence?

Overall, communication was studied or explored as one of a number of possible factors or variables predicting or impacting COVID-19 NPI adoption or adherence. Only one study focused exclusively on the effect of communication on NPI: a longitudinal (two waves of data) analysis explored how aspects of messages affected stay-at-home behaviour over time [31]. By their nature, qualitative studies tended to inductively explore themes (e.g. enablers and barriers) that explained adherence (or non-adherence) to NPIs (e.g. [33,35,38])). Of the five observational (survey) studies, two [27,28] looked at whether conspiracy theories predict NPI adherence, also looking at how conspiracy theories are influenced by communication (channels or sources). Two observational survey studies [29,30] and the one mixed-methods study [39] looked at general or multiple factors predicting NPI adherence, including communication.

Most studies looked at multiple communication channels (media) within one study, including traditional and social media and word-of-mouth communication (e.g. from friends, family and employers). One study [28] focused on public broadcast news (the BBC), and another focused on traditional media [29]. One study [35] explored focused on one form of communication—official communications sent out by programme implementers and stakeholders (e.g. universities and local government) to encourage engagement with COVID-19 testing interventions. Two studies [27,29] conducted separate analyses on specific forms of media (e.g. TV and newspapers). Most (*n* = 8) studies [27–29,33,34,36–38] looked at adherence to multiple NPIs (including, and especially in qualitative studies, generic ‘protection measures’). One study [24] looked specifically at contact tracing app use. Two studies [31,39] looked specifically at quarantine (staying at home). Two studies [32,35] looked at engagement in community COVID-19 testing programs. Most studies looked at NPI adherence in the general UK public (but not with fully representative samples), with two studies focused on local or regional NPI adherence in England [32,35]. Two studies [34,35] focused on diverse ethnicities, and that tailored communication using targeted messages in multiple languages to specific ethnic communities was important for adherence. Very few studies focused on message content and framing. One study [31] specifically explored how aspects of messages related to time spent at home.

### (b) What is the evidence for the effectiveness of communication strategiesin non-pharmaceutical interventions adoption or adherence?

Among the observational studies, evidence of the effectiveness of communications on NPI uptake and adherence was mixed. Two studies [29,30] found that the communications studied had a positive effect on NPI adoption or adherence. One study [28] found no statistically significant effect–between conspiracy beliefs or news source and basic health-protective behaviours. Finally, two studies [27,31] found mixed effects of communication on NPI adoption and adherence. Among the qualitative studies, evidence of the effectiveness of communication on NPI adoption and adherence was also mixed. Two studies [33,39] discussed how communication served both as enablers and a barrier to adherence in their studies. For example, one study [39] found that government communication could have both positive and negative impacts on adherence. Four studies [32,34,36,38] identified only, or focused, on ways in which ineffective communication served as a barrier to adherence (or as a factor in non-adherence). Two studies [35,37] identified only, or focused on, the ways in which effective communication served as enablers to adherence.

In terms of the effect of the type of media, two studies found that engagement with traditional media (e.g. TV and radio) was associated with greater adherence to NPIs [27,29], whereas one study found the use of social media to be associated with lower adherence to NPIs [27]. Two studies [28,30] found no statistically significant effect of news sources on adoption or adherence to NPIs. One found that official sources, including political parties and medical authorities led to higher use of a contact tracing app [30]. One study found that friends and family (word-of-mouth communication) had a small negative effect on adherence [27]. The main themes identified in this review, related to the characteristics of effective communication were clarity and consistency, trust and control. These are summarized in table 2.

**Table 2.** Principles for effective public health communication to influence health behaviour.

theme	findings summary	implication	studies
trust	low trust in government was associated with low adherence to behavioural public health interventions (NPIs)	information should be conveyed by trusted sources (e.g. health authorities)	*N* = 10[23–25,27,29,31–35]
clarity and consistency	too many (often conflicting, unclear) messages were seen as a barrier to adherence (causing ‘alert fatigue’/information overload)	information should be conveyed clearly, and mixed messages should be avoided	*N* = 9[25,27,29–35]
control	messaging focused on supporting autonomy, or being authoritative (but not inducing ‘control aversion’) was associated with higher adherence	communication should strike a balance between being authoritative but avoiding language seen as controlling (e.g. ‘you must’)	*N* = 5[23,27–29,33]

#### (i) Clarity and consistency

The most common feature of effective communication identified was clarity and consistency. Overall, nine studies (one observational, seven qualitative and one mixed methods) [25,27,29–35] had findings which related to this theme. Six studies [29,32,33,37–39] identified message clarity and clear guidance as important. Five of these studies were qualitative, and so clarity of communication (or lack thereof), was defined by participants themselves (i.e. they perceptions of the how clear information was linked to adherence) [32,33,37–39]. One observational study measured quantitatively people's ratings of clearness of information and ‘consistency of instructions and recommendations’ finding that these were significantly positively related to protective behaviours [29]. Four studies [29,33,36,38] identified consistent messages as being important. Studies suggested that participants felt ‘mixed messages’ [33,36], a lack of transparency [35] or inadequate communication of scientific uncertainty [34] led to confusion and in some cases non-adherence to COVID-19 guidance or rules. One study [36] found that too many, often conflicting, messages generated ‘alert fatigue’ or information overload and were seen as a barrier to adherence. Three studies [33,36,39] discussed how the communication of potentially ambiguous messages, rules and terms (e.g. ‘stay alert’ and ‘essential’) were open to interpretation and could therefore be a barrier to adherence.

### (c) What evidence is there on the psychosocial determinants of non-pharmaceutical interventions adoption or adherence?

The evidence on psychosocial determinants was also heterogeneous, with studies using different psychological or behavioural constructs or frameworks. For example, two included studies specifically looked at the relationship between communication, conspiracy beliefs about the origins and spread of COVID-19 (e.g. that it was created or sanctioned by e.g. governments or the pharmaceutical industry) and NPIs. Findings were conflicting, with one study [28] finding that conspiracy beliefs were not statistically significantly associated with basic health-protective behaviours, but the other [27] found a strong negative relationship between conspiracy beliefs and engagement in all health-protective behaviours.

### (i) Trust

The most common psychosocial factor was *trust*, being identified in 10 studies [28–30,33–39]. One prevalent theme (*n* = 6 studies) was that low trust in government resulted in lower adherence to NPIs or protective behaviours [30,33,36,38,39] or to higher belief in conspiracy theories [28]. Conversely, higher trust in medical or health professionals or authorities was associated with higher adherence to NPIs [30,33]. Two studies [35,37] discussed the importance of generally ‘trusted’ or credible sources, for example to engagement in COVID testing [35] on parents’ adherence to various NPIs.

### (ii) Control

Five studies explored the role of control on adherence [28,31,33,34,37]. Legate & Weinstein [31] provided the deepest treatment of the impact of control. Respondents were asked, during the first lockdown (March-May 2020), the extent to which they felt messages were controlling (e.g. ‘conveyed harsh legal consequences of not staying at home’) or autonomy supportive (e.g. ‘provided choices around how to make staying at home work for me’). The study found that autonomy-supportive messages, which framed the behaviour (staying at home) as being in line with individuals beliefs or values and which gave people a sense of choice, encouraged people to spend more time at home. Messages containing language perceived as ‘controlling’ (e.g. ‘you must’ and ‘you should’) were associated with less time spent at home. McNulty *et al.* [27] also discussed their findings in terms of the risks of poorly communicated rules leading to ‘control aversion’. However, whereas controlling language was seen as a potential barrier to adherence, authoritative communication—from sources perceived as official (e.g. national news, official government, or Prime Ministerial announcements) or including mandated messages (i.e. communicating legal requirements) predicted higher adherence to NPIs in three studies [29,31,37].

## 4. Discussion

Health communication, as a ‘meta-intervention’, plays an important role in the effectiveness of many, if not all NPIs, as well as the effectiveness of pharmaceutical interventions. It may, for example, help support the correct use of masks or reduce vaccine hesitancy. However, determining to what extent communication is effective in increasing the adoption of, or adherence to, NPIs, is challenging, for many reasons. Communication is itself a multi-faceted construct making it difficult to ‘isolate’ the impact of any one type or strategy of communication, and to disentangle communication from policy and law. This is especially the case in a public health emergency where rapidly changing information is being transmitted to the public about complex, evolving science, from numerous different sources. Indeed, ‘alert fatigue’ (information overload) has been identified as a barrier to adherence [36].

Notwithstanding these issues and difficulties, this review identified a small body of generally high-quality evidence, particularly obtained through qualitative studies, showing that communication was an important factor in NPI adherence and non-adherence. Overall, communication was good enough to ensure adherence to NPIs was high [3,4,27–29,33,36–39] although included studies also identify the characteristics that led to non- or less-rigorous adherence. This review provides additional evidence, using COVID-19 as a new example, of the importance of key features of effective communication in public health interventions: (i) *information should be conveyed clearly,* (ii) *conflicting (mixed) messages should be avoided*, (iii) *information should be conveyed by trusted sources (*e.g. *health authorities) and* (iv) *communication should strike a balance between being authoritative but avoiding language seen as controlling (*e.g. *‘you must’)* (table 2).

Thus findings here serve to reinforce a large body of risk communication literature across public health (and from environmental health) which also emphasizes that clarity, consistency using trusted information sources and avoiding controlling language are key features of effective communication, designed to encourage adherence or behaviour change (e.g. [1,7,40–46]). Although these are not novel findings, it is a strength of this review, that existing findings usable from non-COVID research is borne out, since it helps to draw out generalizable principles usable for future public health crises.

At the same time, this review identified some limitations and omissions which raise additional questions that warrant further research. We draw out some of these key lessons and implications below. First, there is a need for more empirical research, particularly quantitative research that focuses specifically on communication, as an intervention or strategy. The studies meeting our inclusion criteria did provide useful insights into how communication can be effective, by ensuring it is clear and avoiding mixed messages, using trusted sources. However, a greater focus on communication as a facilitator or barrier to NPI adherence, also controlling for relevant confounders, allows a deeper analysis of some of the psychosocial determinants of its effectiveness (e.g. [31])

There is a need for more studies that actually measure *behaviour* (e.g. the act of adhering) as an outcome, rather than intentions or willingness to adhere. However, there were a number of studies, some of which were well-designed (including RCTs or experimental studies) that were excluded because they only measured the impact of communication on intention or willingness to adopt or adhere to given NPIs. For example, one study found that after using an interactive web-based intervention participants subsequently reported intentions to increase their frequency of infection control behaviours [47]. Future reviews may seek to adopt a broader scope, looking also at the impact of communications on intentions—and perhaps also knowledge and attitudes—related to COVID-19. Nevertheless, while the study of intentions can help to support understanding of the motivations for behaviour, the inclusion of behavioural measures in future studies would further help to confirm these associations and bridge concerns about the intention-behaviour gap [48].

There is a need for longitudinal studies measuring the impacts of interventions on measured behaviour over different time points. This is important because it would help to determine which strategies and interventions have the most enduring impacts, as well as how people's behaviour may change over different stages of a crisis. There may also be benefits to replicating the current review in future to explore whether those studies initially looking at behaviour were subsequently followed up to explore such issues as whether, and which, infection-reducing behaviours (e.g. mask wearing and hand hygiene) were sustained and for how long. Further, multi-method approaches should be adopted to ensure strong evidence on not only how people receive information but how they interpret it and why. For example, including the use of public/citizen panels that could provide real-time evidence of communication effectiveness.

Regarding the UK specifically, more research is needed on ethnicity, diversity and inclusion, such as how best to tailor communications specifically to ethnic minority groups in regard to encouraging and supporting engagement with NPIs. One of the key challenges here would be accounting for the role that cultural and political factors, including national, ethnic and socio-political contexts, play in influencing all aspects of health risk communication (channels, sources, messages and audiences) [49]. Future evidence reviews might also seek to look at additional countries or take an international perspective to support these understandings.

In the UK, government leaders and spokespeople played a prominent role in regularly relaying information, advice and key announcements during the crisis [7]. Some studies have indicated that different leadership styles and strategies had a bearing on risk perceptions along with intentions to engage in safety-related behaviour in relation to COVID-19 for example [50]. However, this specific feature of pandemic communications did not figure as a primary focus of studies meeting criteria for inclusion in this review. Further research investigating the behavioural impacts of leadership communication is therefore warranted to support its use as a focal crisis response tool by officials.

This review highlights the importance of qualitative study findings in supporting research and policy understandings of the use and effectiveness of communication for NPI uptake and adherence. For example, it provided evidence that, from the perspectives of those receiving and interpreting guidance, the way it was communicated may have been insufficiently clear, overly complex or frequent, was perceived as inconsistent or conflicting [32,33,37–39]. However, the incorporation of advice arising from qualitative research evidence can sometimes meet policy resistance especially when viewed in relation to questions of ‘research bias’ and attention to the ‘hierarchy of evidence’ commonly attributed to randomized and controlled quantitative studies. This view can then limit the capacity of qualitative research to inform public health policies and practice. This review indicates that framing the contribution of studies in terms of ‘research quality’ is a useful way to bridge such concerns. It also highlights that the use of well-designed studies employing research panels to obtain qualitative data can offer valuable support to policy understandings of public perceptions (of both the risks and communication practices and impacts) as a crisis emerges and unfolds [51,52].

There were notably few studies that met all of our inclusion criteria, that is: which included a focus on communication (as an independent variable, predictor, mediator or factor); looked at behaviour (i.e. NPI uptake or adherence as a dependent variable or outcome); and which were based/focused on, or included data on the UK. As such there is an overall need for more research, especially RCTs or high-quality (low risk of bias) observational studies, which were otherwise found to be broadly lacking. This may be considered somewhat surprising given the unprecedented volume of research studies conducted in response to COVID-19 as well as the significant allocation of research funds made available through responsive calls in response to the crisis. In the event, besides the predilections of researchers to certain research frameworks and paradigms, many studies were understandably largely fragmented and designed and conducted at short notice in light of the emergency and thereby often limited in focus. Notwithstanding that there are many studies undertaken in response to the crisis yet to be published, this review indicates there is a need to develop ‘crisis ready’ research frameworks or protocols prior to future emergencies to ensure that the most pertinent knowledge is rigorously obtained.

In conclusion, despite the limited volume of evidence, and the need for more higher quality research in future, the studies included in this review suggest that health risk communication can have significant or important impacts on adoption of or adherence to public health behavioural interventions, with more effective communication shaped by the type, clarity and framing of the message and the messenger and communication channels used. Although findings from this review are specifically drawn from the UK, some of its key lessons and implications may be of broader relevance, specifically: the need for clear, consistent and trustworthy communication as facilitators to NPI adherence and the need to develop rigorous and comprehensive behaviourally focused research protocols in advance of future public health challenges. Put simply, poor communication can negatively affect adherence to public health behavioural interventions, whereas good communication can positively affect it.

## Data Availability

This is an evidence review of publicly available previously published studies. The data are provided in the electronic supplementary material [53].
